# Binary classification with fuzzy logistic regression under class imbalance and complete separation in clinical studies

**DOI:** 10.1186/s12874-024-02270-x

**Published:** 2024-07-05

**Authors:** Georgios Charizanos, Haydar Demirhan, Duygu İçen

**Affiliations:** 1 Mathematical Sciences, School of Science, RMIT University, La Trobe St, Melbourne, 3000 Victoria Australia; 2 Department of Statistics, Hacettepe University, Çankaya, Ankara, 06800 Ankara Türkiye

**Keywords:** Clinical studies, Binary classification, Fuzzification, Fuzzy logistic regression, Separation, Class imbalance

## Abstract

**Background:**

In binary classification for clinical studies, an imbalanced distribution of cases to classes and an extreme association level between the binary dependent variable and a subset of independent variables can create significant classification problems. These crucial issues, namely class imbalance and complete separation, lead to classification inaccuracy and biased results in clinical studies.

**Method:**

To deal with class imbalance and complete separation problems, we propose using a fuzzy logistic regression framework for binary classification. Fuzzy logistic regression incorporates combinations of triangular fuzzy numbers for the coefficients, inputs, and outputs and produces crisp classification results. The fuzzy logistic regression framework shows strong classification performance due to fuzzy logic’s better handling of imbalance and separation issues. Hence, classification accuracy is improved, mitigating the risk of misclassified conditions and biased insights for clinical study patients.

**Results:**

The performance of the fuzzy logistic regression model is assessed on twelve binary classification problems with clinical datasets. The model has consistently high sensitivity, specificity, F1, precision, and Mathew’s correlation coefficient scores across all clinical datasets. There is no evidence of impact from the imbalance or separation that exists in the datasets. Furthermore, we compare the fuzzy logistic regression classification performance against two versions of classical logistic regression and six different benchmark sources in the literature. These six sources provide a total of ten different proposed methodologies, and the comparison occurs by calculating the same set of classification performance scores for each method. Either imbalance or separation impacts seven out of ten methodologies. The remaining three produce better classification performance in their respective clinical studies. However, these are all outperformed by the fuzzy logistic regression framework.

**Conclusion:**

Fuzzy logistic regression showcases strong performance against imbalance and separation, providing accurate predictions and, hence, informative insights for classifying patients in clinical studies.

**Supplementary Information:**

The online version contains supplementary material available at 10.1186/s12874-024-02270-x.

## Background

In medical studies, patients are often classified into two groups based on a set of predictors. Diagnosis of medical conditions is one of the most common applications of binary classification in medicine. Common binary classification methods include machine learning, deep learning, and various approaches to logistic regression, such as classical, Bayesian, or fuzzy logistic regression. Predictors describe certain patient characteristics in relation to a medical condition and help estimate the probability of a patient having a certain medical condition ($Y=1$) or not ($Y=0$). Precise prediction of such binary outcomes is imperative, as incorrect classification may lead to misleading conclusions on how effective certain treatments are towards a targeted condition [1, 2]. It may also affect government policy on regulating these treatments or lead to resource and ethical complications.

Two core problems with binary classification that may cause such challenges are the issues of class imbalance and complete separation [3, 4].

Firstly, we focus on the problem of class imbalance. Class imbalance occurs when the frequency in the binary responses that classify a patient as having or not having a medical condition is greater towards one instance rather than equally distributed across both. For example, for a classifier created to diagnose breast cancer patients given a specific diagnostic procedure, the number of subjects diagnosed as having the condition will be significantly lower than those without breast cancer. This creates a considerable class imbalance in the binary responses of the clinical dataset. Due to this imbalance, a model may become sensitive towards predicting the dominant class, hence providing biased results towards the Benign class. It is essential for statistical models to produce strong binary classification performance under such settings of class imbalance, ensuring a stronger understanding of the true association between predictors and the probability of being classified as having the medical condition.

Secondly, complete separation occurs when a set of predictors used in predicting a medical diagnosis, such as patient demographic information, medical history, and associated variables, perfectly cluster the binary responses. Separation causes over-fitting in the model [5], which learns to predict a medical diagnosis based on the relationship between a patient being classified as having a condition or not and a subset of medically relevant variables. We see the impact of over-fitting in the significance tests of the classical logistic regression. Over-fitting inflates the performance measures and reduces the test performance of machine learning methods in classification tasks [6, 7]. For example, we expect cancer diagnosis to be more frequent in older individuals as the human body becomes more vulnerable with age [8]. In a dataset of patients of all ages, the age group variable would perfectly separate the classification of having cancer in older people. Hence, this relationship would become dominant in the classification model due to complete separation. The most common way of detecting such clustering is using scatter-plots between sets of predictors and the binary responses [9]. However, this is not always practical in situations with a large number of predictors or large sample sizes, making scatter plots hard to interpret.

The literature encompasses some limitations on the problems of class imbalance and complete separation. Kumar et al. [10] address the problem of imbalanced datasets in clinical studies by exploring several methods, such as random oversampling, synthetic minority oversampling, adaptive synthetic sampling, and fuzzy nearest neighbors. The results indicate sufficient classification performance in handling imbalance problems due to high precision and F1 scores. However, these scores could be inflated due to separation, and the lack of considering sensitivity and specificity measures makes it difficult to determine if a method is truly robust toward class imbalance.

Specificity and sensitivity measures can be used to determine whether a model is influenced by class imbalance or not. This is achieved by observing if there is a large variation between these measures. For example, consider a dataset that has $95\%$ of binary instances in the $Y=0$ class. This indicates that the dataset is significantly imbalanced towards $Y=0$. If the classifier is impacted by this imbalance, we would observe a high specificity score (potentially close to 1.000) with a low sensitivity score. On the other hand, given that complete separation can lead to over-fitting, the classifier only learns from a subset of dominant predictors in the data, meaning that the impact on classification performance can be seen through the presence of very large performance scores across all measures used in the study [9, 11]. Consider the example of cancer diagnosis based on age; the classifier mostly learns from the relation of age with a cancer diagnosis and, hence, produces performance outcomes very close to 1.000, equally seen across specificity, sensitivity, F1, and Mathew’s correlation coefficient (MCC) scores. The classifier fails to establish what other conditions may impact the classification of cancer in patients, which reduces the accuracy of the classification when new data is induced in the clinical study or the classifier is used with additional observations outside the main sample.

Yazdani et al. [12] propose three approaches, namely Naive Bayes, decision tree, and artificial neural networks (ANN), for diagnosing breast cancer in patients. These approaches are applied on a dataset with an imbalance of 70% towards the $Y=0$ group. ANN achieves the highest accuracy (0.945), followed by the decision tree (0.932) and Naive Bayes (0.901). Sensitivity and specificity are also considered for the ANN model, with sensitivity at 0.962 and specificity at 0.864. The authors employ generalized accuracy scores to assess classification performance, which does not provide a complete assessment of classification performance across the full length of a confusion matrix. As such, there is no consideration of how well these three algorithms are able to classify breast cancer diagnosis in patients irrespective of the presence of imbalance or separation in the available clinical data. Assessing imbalance and separation would offer greater insights into how well the model detects the minority class under imbalance and if separation is inflating performance scores due to over-fitting from a subset of predictors in the data. The simultaneous assessment of both imbalance and separation is a crucial step in assessing the performance of any classifier. Even if a classifier showcases strong results across a set of performance measures under imbalance, these results could be artificially inflated in the presence of separation. Li et al. [13] only consider accuracy when diagnosing breast cancer, even though their data set has an imbalance of 70% towards the $Y=0$ group. Jenni et al. [14] propose using machine learning methods to classify patients regarding the presence of breast cancer. The authors consider regularized general linear models (GLMs), support vector machines (SVMs) with a radial basis function kernel, and single-layer ANN. While these models showcase strong classification performance (sensitivity $0.970-0.990$, and specificity $0.850-0.940$), there is no consideration of complete separation and whether the separation would inflate these performance scores.

On the other hand, Guo et al. [15] focus specifically on the issue of class imbalance, proposing a new method called imbalanced logistic discrimination to improve the classification performance of logistic discrimination for the diagnosis of breast cancer. They evaluate classification performance using specificity, sensitivity, F1 score, and G-means. Their model shows high performance scores, with a sensitivity of 0.734, a specificity of 0.869, and an F1 score of 0.624. At first glance, these appear to be strong results with a small variation between sensitivity and specificity. However, sensitivity is relatively low here for a clinical study, with only $73.4\%$ of the patients with a medical condition classified correctly. Moreover, Chicco and Jurman [16] consider various machine learning models to predict survival rates in heart failure patients with a 68% imbalance towards $Y=0$, such as random forests, decision trees, SVM, and extreme gradient boosting. The class imbalance in the data is considered, and the authors utilize a comprehensive list of classification performance measures, such as MCC, receiver operating characteristic (ROC) curves, the area under the curve (AUC), sensitivity, specificity, and F1 score. However, despite focusing on classification performance against imbalance, only the random forests method achieves strong classification performance given the measures considered, with a lack of variation between specificity and sensitivity scores. Qanbar and Algamal [17] improve SVMs against the imbalance problem in classification with large data. In contrast, all other machine learning methods indicate the impact of class imbalance on performance by producing lower accuracy scores or showing large variations between sensitivity and specificity scores. However, these results may be inflated by separation issues, while the authors do not offer an analysis of classification performance against complete separation or if separation exists in the data. Analysis targeted at separation problems in combination with imbalance offers insights into the true magnitude of classification performance for these machine learning methods.

Complete separation can be harder to detect when using a percentage accuracy score or an insufficient set of performance measures [10, 12, 13, 18]. Hence, while there might be an indication of strong performance under class imbalance settings, these results could be artificial. Cook et al. [19] highlight the importance of considering separation and its over-fitting impact in a more general family of classification models, namely, multinomial logistic regression models. Zorn [18] considers the separation problem in statistical models and proposes using Firth’s [20] penalized likelihood approach against separation in statistical models. Zorn [18] does not take class imbalance into account in addition to separation. Suleiman et al. [21] implement a Bayesian logistic regression in health management data using Firth’s [20] penalized likelihood against separation. However, they do not consider class imbalance along with separation since it does not exist in their data. Masournia et al. [22] investigate the causes and impacts of separation in logistic regression. Crisman-Cox et al. [23] consider separation in strategic choice models. Recently, Charizanos et al. [24] deal with both separation and class imbalance problems in credit card fraud detection data. Regarding the literature, separation is a core problem in binary classification tasks under any type of modeling. Hence, researchers need to consider this very common problem when validating model performance. Kumar et al. [10] discuss the imbalance issue in great length, but there is no consideration of complete separation and how it could inflate performance scores. Similarly, Yazdani et al. [12] and Li et al. [13] do not mention complete separation while using simple percentage accuracy scores, which can be artificially inflated due to separation in the data. On the other hand, Guo et al. [15] and Chicco and Jurman [16] utilize a better set of performance measures, focusing on sensitivity, specificity, F1 score, ROC, and AUC. While this larger variety of performance measures can offer targeted insights, there is no direct consideration of these scores and their association with complete separation. This could be achieved by investigating the presence of large scores across multiple performance measures and giving different subsets of predictors that could be causing complete separation in the data.

The following motivating example demonstrates the impact of class imbalance and separation issues on classification performance.

### Motivating example

**Fig. 1:**
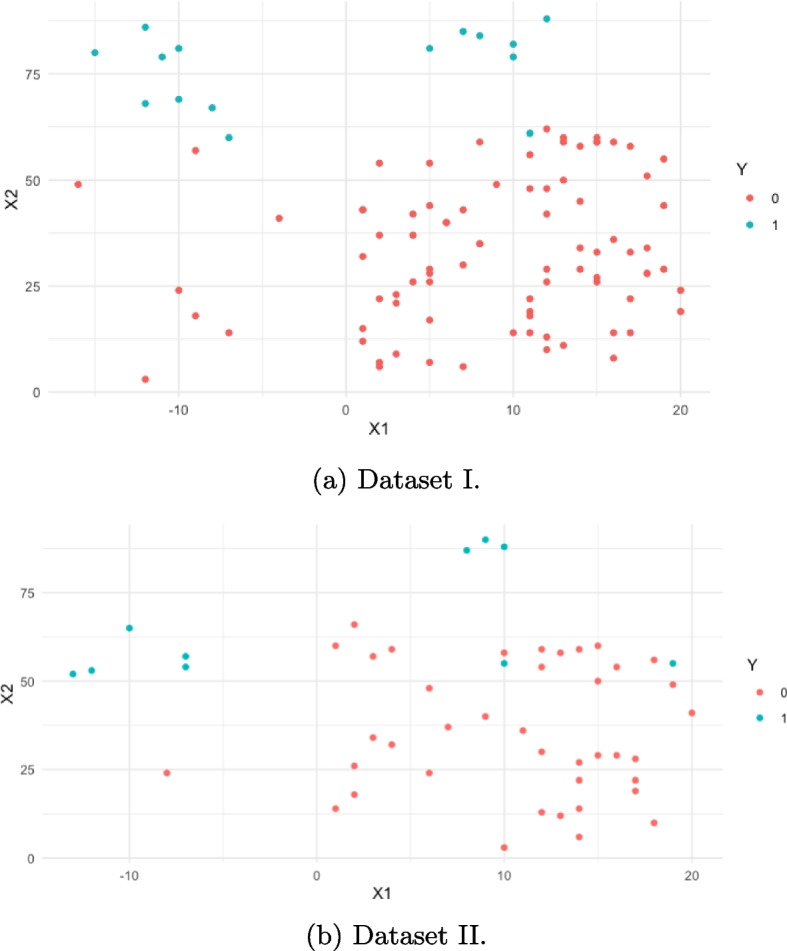
Scatter plot of X1 and X2 in the breakdown of Y for Datasets I and II

We arbitrarily created two artificial datasets from the normal distribution, namely Dataset I and II, and fitted 3 logistic regression models with these datasets to demonstrate the combined impact of class imbalance and complete separation. Dataset I is used as the main sample, and Dataset II is used as new data for predictions. Descriptive information on the datasets is given in Table 1. Both datasets have a high imbalance towards the $Y=0$ class. *X*1 takes both negative and positive values and has a significant impact on the response variable but does not create separation. However, *X*2 is all positive and mimics a predictor such as age that creates separation. To create separation, the observations that correspond to the $Y = 1$ (or $Y = 0$) class need to be more homogeneous than those that correspond to the $Y = 0$ (or $Y = 1$) class with a significantly different average. This is what we create in X2. We have $Var(X2|Y=1) = 0.3\cdot Var(X2|Y=0)$ while $E(X2|Y=1) = 2.33\cdot E(X2|Y=0)$. So, older individuals who have the condition have considerably smaller variation; hence, having the condition at higher ages is more certain. However, to create a significant independent variable not creating separation, the variation between the $Y = 1$ (or $Y = 0$) class and the $Y = 0$ (or $Y = 1$) class needs to be smaller with a large relative average between the classes. So, for *X*1, we have $Var(X1|Y=1) = 1.6\cdot Var(X1|Y=0)$ with $E(X1|Y=0) = -5.8\cdot E(X1|Y=1)$. In this way, X1 will be significant, but since one of the classes is not considerably more homogeneous than the other, it will not overtrain the model toward the impact of any of the classes. The separation created in Dataset I with these data generation settings is also evident in Fig. 1a. Dataset II mimics new data with a slightly lower magnitude of separation as in Fig. 1b.

**Table 1:** Datasets for assessing class imbalance and separation

			Average $\varvec{X1}$ for		Average $\varvec{X2}$ for	
Dataset	Sample size	Imbalance	$\varvec{Y=0}$	$\varvec{Y=1}$	$\varvec{Y=0}$	$\varvec{Y=1}$
I	100	85%	8.7	-1.5	32.9	76.6
II	50	80%	9.6	0.1	35.9	65.6

Imbalance column shows the proportion of $Y=0$ class

First, we fit a model with only *X*1 predictor, namely Model I. The confusion matrix for this model is given in Table 2. Since $Y=0$ class has the majority due to 85% class imbalance, 11 observations are misclassified to $Y=0$ class. The impact of class imbalance is clear in this model. Now, we add *X*2 to Model I to create Model II and the confusion table given in Table 2. Since *X*2 creates separation, Model II learns from *X*2 excessively and compensates for the impact of class imbalance, resulting in reduced misclassification of $Y=1$s to only 1 observation. Although this looks acceptable regarding classification performance, Model II suffers from the over-fitting issue. Next, we create predictions for Dataset II using Model II to investigate the use of a classifier with a new dataset, namely the impact of over-fitting. From the last confusion matrix in Table 2, Model II misclassifies 60% of $Y=1$s in the new data due to the over-fitting issue. Given the presence of complete separation, Model II now mostly learns from a subset of predictors in the data, which perfectly separate the binary responses. Thus, Model II is unable to learn from new data introduced into the study.

**Table 2:** Confusion matrices of the classifier on the three key binary classification examples

	Predicted Y=0	Predicted Y=1
Model I		
Actual Y=0	82	2
Actual Y=1	11	4
Model II		
Actual Y=0	83	1
Actual Y=1	1	14
Model II with Dataset II		
Actual Y=0	39	1
Actual Y=1	6	4

Table 3 shows the performance scores corresponding to the confusion matrices from Table 2. Given the distinct imbalance setting under Model I impacts the false negative rate, a much lower sensitivity score of 0.267 is observed with an increased specificity of 0.976. This large variability between these scores indicates that the models are impacted by class imbalance, allowing for an efficient detection of such issues. Once separation is induced in Model II, we see that these scores increase to almost perfect classification performance across all four measures. These results can be misleading, as researchers may interpret them as accurate classification performance despite imbalance issues. However, they are artificial due to over-fitting caused by separation. As such, when Model II is applied to Dataset II, we see a performance reduction with much lower sensitivity, F1, and MCC scores. This is because the model now imposes its over-learning on the classification of new data and fails to capture the patterns associated with the new data.

**Table 3:** Calculated classification performance measures for the given examples

Example Model	Sensitivity	Specificity	F1	MCC
Model I	0.267	0.976	0.381	0.365
Model II	0.933	0.988	0.933	0.921
Model II with Dataset II	0.400	0.975	0.533	0.500

As is also seen in the motivating example, to achieve a holistic insight into the true performance in such classification tasks, we need to consider the problems of separation and imbalance simultaneously. Consequently, it is more important to have a classifier that is influenced by neither class imbalance nor separation in the data. To the best of our knowledge, the works in the literature do not consider the problem of complete separation, individually or simultaneously, with class imbalance for clinical studies.

Fuzzy logistic regression introduced by Charizanos et al. [9] offers greater classification accuracy against class imbalance and complete separation issues. The method induces a level of fuzziness in the data using triangular fuzzy numbers for coefficients, model inputs, and outputs. Moreover, a sufficient set of performance measures should also be employed to provide a more thorough assessment of how the model behaves under class imbalance and complete separation. Charizanos et al. [9] recommend the use of sensitivity, specificity, F1 score, and MCC. A confusion matrix of the classification results is also considered.

The main motivation of our study is to propose a solution involving fuzzy logistic regression to tackle the limitations identified in the literature. The objectives of our study are as follows: Initially, we identify the extent of separation and imbalance that exists across twelve different clinical study datasets, which are commonly utilized in studies involving classification methods. This is a crucial step that helps target our analysis of classification performance and benchmarking studies from the literature. We then aim to implement the fuzzy logistic regression framework to showcase strong classification performance across a comprehensive set of performance measures. Thirdly, we assess classification performance against class imbalance and complete separation, considering the clinical datasets’ various settings and scenarios.

This study has the following contributions:

- The proposed approach showcases strong classification performance under class imbalance conditions by employing the fuzzy logistic regression framework for the majority of clinical studies considered.
- It achieves strong performance against complete separation in the data using the fuzzy logistic regression framework.
- It provides a generalizable methodology for any type of clinical study, irrespective of data complexity. The codes to implement the proposed methodology are readily available: https://github.com/GZanos/Fuzzy-Logistic-Regression.

The Data and methodology section presents the descriptive analysis of the utilized datasets and the overall methodology of the proposed framework. The Application section details the proposed method’s application, focusing on the identified limitations and benchmarking against other methods. The last section presents a general discussion and conclusions.

## Data and methodology

### Datasets and imbalance

We utilize 12 clinical study datasets in this study. These are all binary classification datasets and are related to various clinical study types, such as breast cancer, hepatitis, diabetes, liver disorders, heart disease, fertility disease, and Parkinson’s disease. Table 4 shows the sample size, *N*, the number of predictors, *k*, and the level of imbalance in the binary responses. The fertility dataset has the smallest sample size of just $N=100$, while the diabetic retinopathy debrecen dataset is the largest with $N=1151$. The liver disorders dataset has just 7 predictors, while the Parkinson’s disease dataset has the largest set of predictors. The datasets provide a good variety of different sample sizes and sets of predictors for the generalizability of the results.

**Table 4:** The sample size, number of predictors, and level of imbalance for utilized datasets

Dataset Name	$\varvec{N}$	$\varvec{k}$	Imbalance (Y=0 | Y=1)	Separation
Breast Cancer [25]	286	9	70% | 30%	No
Breast Cancer Wisconsin (Prognostic) [26]	198	34	76% | 24%	No
Breast Cancer Wisconsin (Diagnostic) [27]	569	32	63% | 37%	Yes
Hepatitis [28]	155	19	79% | 21%	Yes
Pima Indians Diabetes Database [29]	768	8	65% | 35%	Yes
Liver Disorders [30]	345	7	42% | 58%	No
SPECTF Heart [31]	267	22	21% | 79%	Yes
Fertility [32]	100	10	12% | 88%	No
Diabetic Retinopathy Debrecen Dataset [33]	1151	20	47% | 53%	Yes
Breast Cancer Coimbra [34]	116	10	44% | 56%	No
Parkinson’s Disease Classification [35]	756	754	25% | 75%	Yes
Heart failure clinical records [36]	299	13	68% | 32%	No

In terms of class imbalance levels, there are six datasets with at least $70\%$ imbalance towards one of the binary classes. These datasets are considered to be significantly imbalanced. There are three datasets with moderate levels of imbalance, which is between $60-70\%$ towards one class, and three datasets are fairly balanced.

### Separation detection in data

We observe large or infinite values for maximum likelihood estimates to detect separation as outlined by Kosmidis and Schumacher [37]. Classical logistic regression is first implemented with the given data and the maximum likelihood (ML) estimation for model parameters. Then, the presence of large or infinite ML estimates on the refitted model during ML estimation indicates complete separation in the data, as described by Lesaffre and Albert [38]. This is done by observing the ML estimates across the iterations of the maximum likelihood optimization algorithm. The presence of large ML estimates that deviate significantly from the rest of the values is usually attributed to large coefficient estimates, which offer evidence of the presence of separation in the data [39]. The results of this analysis on the considered datasets are presented in the Separation analysis results section.

### Fuzzy logistic regression

A triangular fuzzy number (TFN), $\bar{A}$, is defined by three values, $a_1<a_2<a_3$, with a triangular fuzzy membership function, $\mu _A(x)$. The interval of the TFN is given by the limit of $[a_1, a_3]$, while the vertex is $a_2$ [40]. The level of symmetry in a TFN is measured based on the distance between $||a_1, a_2||$ and $||a_2, a_3||$. For example, a symmetric TFN has $||a_1, a_2||=||a_2, a_3||$, while in an asymmetric TFN we have $||a_1, a_2||\ne ||a_2, a_3||$. The degree of fuzziness in a TFN is determined by the length of its interval $[a_1, a_3]$. The greater the length of this interval, the more fuzziness is induced in the data. Moreover, the $\alpha$-cut of a TFN, $\bar{A}$, is a set of real numbers defined as $\bar{A}_{(\alpha )}=[x\in {R},\mu _A(x)\ge {\alpha }]$. As such, we have $\bar{A}_{(\alpha )}=[A^L_{(\alpha )},A^U_{(\alpha )}]$, where $A^L_{(\alpha )}=inf[x\in {R},\mu _A(x)\ge {\alpha }]$ and $A^U_{(\alpha )}=sup[x\in {R},\mu _A(x)\ge {\alpha }]$ [40].

The fuzzy logistic regression framework of Charizanos et al. [9] estimates the outcome probability of a binary event as shown in Eq. (1):

1 $$\begin{aligned} \log \left( \frac{\bar{P}}{1-\bar{P}}\right) = \bar{\beta }_{0} + \bar{\beta }_{1}\bar{X_1} + \bar{\beta }_{2}\bar{X_2} + \dots + \bar{\beta }_{k}\bar{X_k}, \end{aligned}$$

where all coefficients $\bar{\varvec{\beta }}=(\bar{\beta }_{j}), j= 0,1,\dots ,k$, along with predictors $\varvec{X}=(X_{i}), i=1,\dots ,k$ are TFNs. Eq. (1) captures the relationship between the fuzzy response variable, $\bar{Y}$, and the fuzzy coefficients and predictors to estimate the fuzzy success probabilities, $\bar{P}\in [0,1]$.

Binary responses are fuzzified into TFNs, such that $y_1<y_2<y_3$ and with a triangular fuzzy membership function $\mu _Y(x)$ [40], by defining $\bar{Y}$ as a function $F:\mathbb {Z}_{2}\rightarrow \mathbb {R}^{'}$ with $\mathbb {Z}_{2}=\{0,1\}$ and $\mathbb {R}^{'}=\{x\in \mathbb {R}|-1\le x \le 1\}$. Then, $F(Y)=[Y-m\cdot \ell \cdot U,Y,Y+m\cdot r \cdot U]$, where $U\sim Uniform(I_{L},I_{U})$ [9]. The value of *m* is the degree of fuzziness induced in the data, while *r* and $\ell$ are to adjust the symmetry of output TFN, and $I_{L}$ and $I_{U}$ are the upper and lower limits.

To estimate the fuzzy coefficients, a pre-defined range of $\bar{I}_{\beta }=[\bar{I^-},\bar{I^+}]$ is taken, and random crisp vectors are generated and ordered within this range, such that $v_k=(x_{1k},...,x_{3N+3,k})$, where $x_{ik}\in {[0,1]}$. Fuzzy vectors $\bar{V}_{k}=(\bar{V}_{1k},...,\bar{V}_{Nk})$ are then extracted from the random set of crisp vectors $v_k$, which are then plugged in as coefficients in Eq. (1) [9].

Then, the optimization measures mean absolute error (MAE), mean squared error (MSE), and root mean square error (RMSE) are calculated between the observed and predicted TFNs corresponding to $\bar{V}_{k}$. This is repeated many times to get a sample of optimization measures. Then, the optimization measure that provides the lowest variation is used as the main optimization measure and the corresponding TFN coefficients are used for prediction.

A fuzzy threshold $\bar{\tau }=(a_{1}, a_{2}, a_{3})$ is selected, and the vertex position of each predicted TFN probability, $\bar{P}$, is classified by comparison against $\bar{\tau }$. Firstly, the expected value of $\bar{P}$, $\mathbb {E}(\bar{P})$ is calculated using Dubois and Prade [40]’s approach. Secondly, the difference between $\bar{P}$ and $\bar{\tau }$ and width, $w(\mathbb {D})$ are estimated. A comparison is made, such that if $\mathbb {E}(\bar{P})<a_{2}$, $\hat{y}_{2}=0$, otherwise, $\hat{y}_{2}=1$. The resulting output includes a set of predicted TFN values for $\bar{Y}$, where the vertex $\hat{y}_{2}$ is equal to either 0 or 1, while $\hat{y}_{1}$ and $\hat{y}_{3}$ points are estimated by $\hat{y}_{1} = \hat{y}_{2} - w(\mathbb {D})$ and $\hat{y}_{3} = \hat{y}_{2} + w(\mathbb {D})$.

### Performance measures

The measures used to assess the classification performance are sensitivity, specificity, F1 score, and MCC, as shown in Table 5. Specificity is defined as the ability of a classifier to identify negative instances correctly, while sensitivity is the ability of a classifier to identify positive cases correctly. The F1 score evaluates the accuracy of a classifier by combining both precision and recall, while MCC evaluates the accuracy of a classifier by taking into account true positive and true negative cases and false positive and negative cases.

**Table 5:** Definitions of performance measures

Metric	Definition
True Positives (TP)	Number of correctly predicted positive instances
True Negatives (TN)	Number of correctly predicted negative instances
False Positives (FP)	Number of incorrectly predicted positive instances
False Negatives (FN)	Number of incorrectly predicted negative instances
Specificity	$TN/(TN+FP)$
Sensitivity	$TP/(TP+FN)$
Precision	$TP/(TP+FP)$
Recall	$TP/(TP+FN)$
F1 Score	$(2 \times \text {Precision} \times \text {Recall})/(\text {Precision} + \text {Recall})$
MCC	$(TP \times TN) - (FP \times FN)/\sqrt{(TP + FP)(TP + FN)(TN + FP)(TN + FN)}$

Sensitivity and specificity provide a comprehensive evaluation of classification against the combined impact of imbalance and separation. When a model is influenced by class imbalance, an increase occurs in either false positive or false negative instances in the confusion matrix, which depends on which class the class imbalance is leaning toward. In turn, this creates strong variability between sensitivity and specificity scores due to significant differences in true positive and true negative rates. For example, a model impacted by a dataset with a significant imbalance towards $Y=1$ will create high sensitivity and low specificity scores because it will result in a high true positive and low true negative rate. However, as shown in the motivating example, the presence of complete separation can artificially inflate classification performance, resulting in lower false positive and false negative rates. This inflates sensitivity and specificity scores to almost one. Utilizing F1 and MCC scores provides further evidence of such inflation and can act as a way to identify over-fitting caused by separation issues.

## Application

First, a separation detection analysis is conducted to determine the datasets that complete separation affects. We then assess the classification performance of fuzzy logistic regression against machine learning methods by employing 10-fold cross validation across all 12 datasets and models considered. This comparative analysis, along with post-hoc significance tests, outlines the performance of the fuzzy logistic regression against machine learning models.

### Separation analysis results

**Fig. 2:**
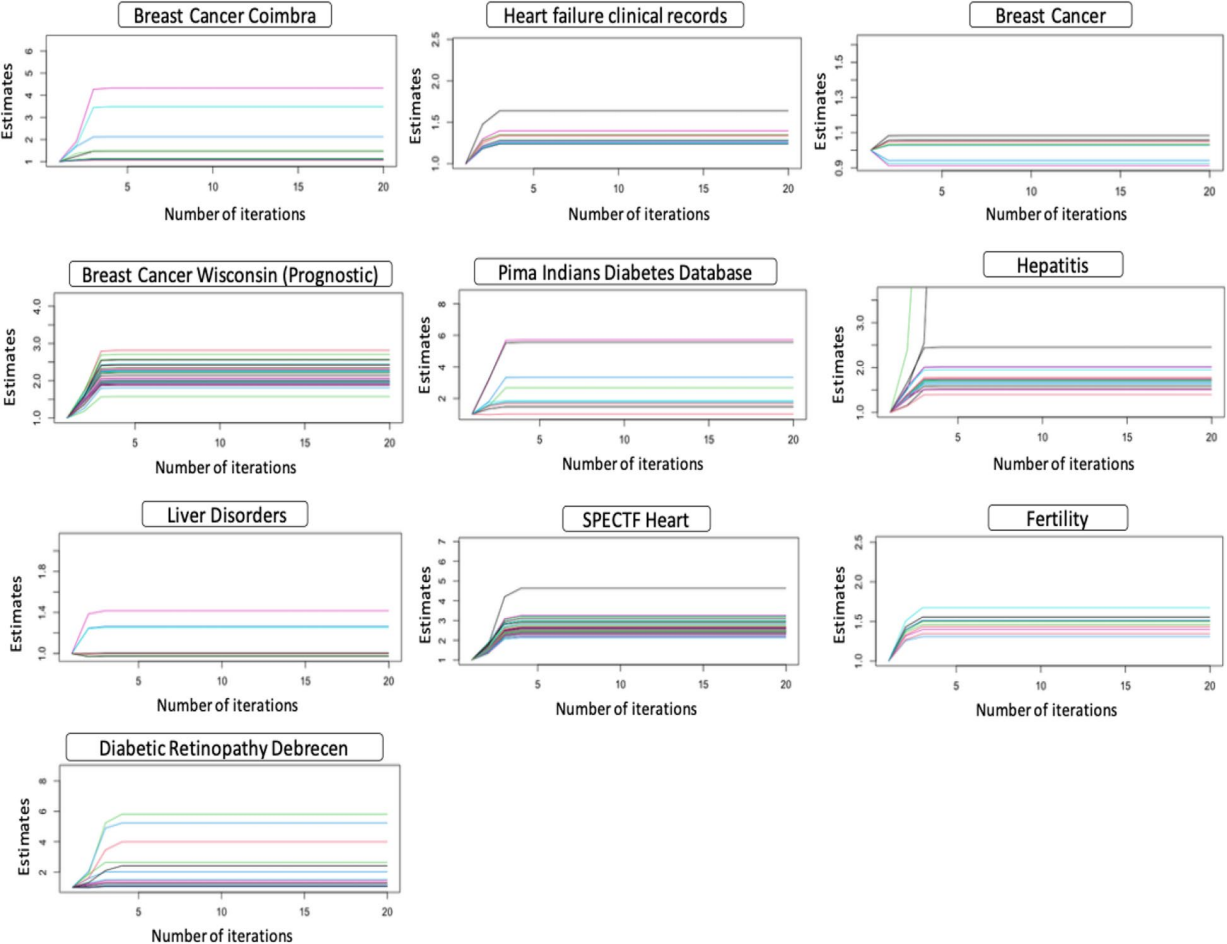
Maximum likelihood estimate for each model parameter for the clinical datasets considered

Figure 2 shows the ML estimates from the logistic regression model through the iterations of ML optimization with all combinations of predictors and binary response variables. Evidence of complete separation is presented as values of maximum likelihood estimates that rapidly increase to infinity or large values that significantly deviate from the rest of the estimates.

We see no evidence of separation for the breast cancer dataset or the breast cancer Wisconsin (prognostic) dataset since no parameter estimate gets abnormally inflated as the number of iterations increases. However, in the hepatitis dataset, estimates of two parameters deviate to infinity, while a third deviates to larger values. Similarly, in the Pima Indians diabetes dataset, two estimates deviate significantly from the rest of the parameter estimates. These present evidence of complete separation in these two datasets. Note that the breast cancer Wisconsin (diagnostic) dataset shows all estimates to infinity due to complete separation in the data. Hence, no plot was produced. Similarly, the liver disorders, fertility, breast cancer Coimbra, and heart failure datasets show no signs of separation since none of the estimates deviate to infinity or large values. However, we see evidence of separation in the SPECTF heart, diabetic retinopathy Debrecen, and Parkinson’s disease datasets.

The results are summarized in Table 4, showcasing the datasets with signs of separation. Overall, 6 out of 12 considered datasets have the separation issue. When considered together with class imbalance 5 out of 12 datasets have both problems.

### Classification performance of fuzzy logistic regression against imbalance and separation and benchmarking with machine learning models

If the imbalance in the data influences the classification performance of a model, we expect to see a notable variation between specificity and sensitivity results. As shown in the motivating example, a dataset imbalanced towards $Y=1$ would result in a sensitivity close to 1 and a specificity close to 0. On the other hand, complete separation creates over-fitting, resulting in extremely high-performance scores, such as values very close to 1.000 or larger than 0.900 for all performance measures.

K-fold cross-validation is a frequently employed method for generalizing model performance. We implement the fuzzy logistic regression framework on 10 folds per dataset with $70-30\%$ split for all 12 datasets. We implement six machine learning models, namely SVM, ANN, KNN, random forest (RF), XG-Boost (XGB), and imbalanced learning (IBL), using the same 10 folds. Furthermore, the same models are applied with and without implementing a SMOTE balancing method on each fold. Sensitivity, specificity, F1 score, and MCC metrics are calculated for each model. Then, the significance of the difference between the performances of the compared models in terms of each metric is tested by using Friedman and Nemanyi tests following Demsar [41] over 12 datasets. This way, we benchmark FLR with machine learning models with and without SMOTE using 10-fold cross-validation and statistical significance tests.

Tables 6 and 7 show the sensitivity and specificity results for FLR and all machine learning methods. FLR shows consistently strong sensitivity and specificity scores, ranging between $0.799-0.937$ for sensitivity and $0.683-0.938$ for specificity. The SMOTE-FLR model shows improvement due to the balancing method, with sensitivity ranging between $0.844-0.995$ and specificity between $0.840-0.994$. There are no instances of large variability between sensitivity and specificity scores for any specific dataset when using FLR methods, which is a strong indicator that the imbalance and separation do not influence FLR implementations. On the other hand, we see a significant impact of imbalance and separation on machine learning models. SVM, ANN, KNN, XGB, CLR, and RF methods show significant variability between sensitivity and specificity for most of the datasets, indicating the impact of imbalance. Applying SMOTE for these machine learning methods seems to notably improve their performance. Their average specificity score across all 12 datasets without SMOTE is 0.602, which increases to 0.668 with SMOTE. Similarly, sensitivity improves from 0.671 to 0.687 with SMOTE for the machine learning methods. However, these scores are lower than those of FLR with and without SMOTE, averaging 0.913 and 0.906 for sensitivity and specificity, respectively. The classical logistic regression (CLR) model produces similar sensitivity and specificity scores as the machine learning methods, with an average of 0.682 and 0.680, respectively, improving to 0.716 and 0.665 when SMOTE is applied.

**Table 6:** Sensitivity results of fuzzy logistic regression against machine learning models

	Without SMOTE							With SMOTE							
Dataset	FLR	SVM	ANN	KNN	RF	XGB	CLR	FLR	SVM	ANN	KNN	RF	XGB	CLR	IBL
Breast Cancer	0.874	0.965	0.911	0.923	0.932	0.868	0.885	0.995	0.669	0.600	0.644	0.741	0.690	0.636	0.741
Breast Cancer Wisconsin (Prognostic)	0.880	0.998	0.967	0.967	0.983	0.945	0.829	0.992	0.741	0.728	0.568	0.743	0.708	0.708	0.743
Breast Cancer Wisconsin (Diagnostic)	0.813	0.982	0.964	0.965	0.976	0.977	0.941	0.995	0.966	0.961	0.939	0.958	0.957	0.930	0.958
Hepatitis	0.799	0.973	0.967	0.968	0.970	0.918	0.901	0.993	0.871	0.720	0.567	0.868	0.785	0.731	0.868
Pima Indians Diabetes Database	0.867	1.000	1.000	0.844	0.872	0.869	0.921	0.911	0.539	0.550	0.569	0.645	0.655	0.552	0.645
Liver Disorders	0.911	0.501	0.510	0.526	0.595	0.625	0.568	0.936	0.635	0.654	0.618	0.731	0.727	0.701	0.731
SPECTF Heart	0.920	0.135	0.000	0.393	0.176	0.362	0.491	0.942	0.755	0.591	0.903	0.750	0.722	0.609	0.750
Fertility	0.937	0.000	0.000	0.000	0.000	0.075	0.025	0.953	0.278	0.265	0.497	0.358	0.380	0.583	0.358
Diabetic Retinopathy Debrecen	0.922	0.759	0.815	0.700	0.718	0.710	0.816	0.923	0.796	0.798	0.660	0.800	0.771	0.829	0.801
Breast Cancer Coimbra	0.929	0.737	0.454	0.429	0.672	0.678	0.725	0.924	0.775	0.676	0.612	0.739	0.720	0.728	0.739
Parkinson’s Disease	0.913	0.014	0.312	0.268	0.366	0.455	0.190	0.926	0.656	0.658	0.621	0.639	0.641	0.808	0.644
Heart failure	0.823	0.905	0.936	0.908	0.915	0.909	0.893	0.844	0.784	0.478	0.481	0.833	0.843	0.777	0.833

**Table 7:** Specificity results of fuzzy logistic regression against machine learning models

	Without SMOTE							With SMOTE							
Dataset	FLR	SVM	ANN	KNN	RF	XGB	CLR	FLR	SVM	ANN	KNN	RF	XGB	CLR	IBL
Breast Cancer	0.914	0.222	0.329	0.222	0.316	0.391	0.379	0.937	0.552	0.561	0.566	0.538	0.544	0.661	0.538
Breast Cancer Wisconsin (Prognostic)	0.844	0.051	0.065	0.021	0.161	0.378	0.585	0.971	0.563	0.549	0.576	0.553	0.560	0.568	0.553
Breast Cancer Wisconsin (Diagnostic)	0.860	0.965	0.917	0.873	0.935	0.960	0.916	0.964	0.966	0.946	0.888	0.967	0.968	0.907	0.967
Hepatitis	0.902	0.199	0.111	0.014	0.328	0.419	0.500	0.954	0.619	0.552	0.631	0.593	0.632	0.521	0.593
Pima Indians Diabetes Database	0.923	0.000	0.000	0.252	0.299	0.274	0.197	0.959	0.782	0.785	0.674	0.711	0.600	0.807	0.709
Liver Disorders	0.876	0.841	0.808	0.810	0.816	0.771	0.793	0.994	0.665	0.652	0.686	0.646	0.671	0.626	0.648
SPECTF Heart	0.847	0.949	1.000	0.848	0.960	0.889	0.825	0.993	0.741	0.688	0.534	0.747	0.737	0.670	0.749
Fertility	0.683	1.000	0.978	1.000	0.985	0.975	0.944	0.891	0.727	0.627	0.532	0.714	0.639	0.566	0.714
Diabetic Retinopathy Debrecen	0.924	0.624	0.704	0.603	0.639	0.670	0.681	0.922	0.594	0.692	0.598	0.540	0.623	0.666	0.540
Breast Cancer Coimbra	0.909	0.723	0.740	0.561	0.758	0.761	0.709	0.931	0.714	0.668	0.503	0.661	0.743	0.687	0.661
Parkinson’s Disease	0.828	0.990	0.874	0.931	0.929	0.871	0.985	0.84	0.532	0.649	0.663	0.721	0.705	0.545	0.719
Heart failure	0.938	0.591	0.372	0.113	0.683	0.661	0.674	0.946	0.715	0.812	0.450	0.831	0.786	0.754	0.831

The results of Friedman and Nemenyi tests for overall and pairwise comparison of the model performance metrics are given in Supplementary Material (SM). Friedman tests for both sensitivity and specificity produce $P<0.001$ with $Q=59.195, F_f=8.476$ and $Q=59.337, F_f=8.511$, respectively, indicating that there is a significant difference between the models’ sensitivity and specificity performances at 5% level of significance. Nemenyi’s post hoc tests, given in Tables S1 and S2 of SM, show that the significant difference is mainly due to the FLR models, which produce much stronger sensitivity and specificity performance. This analysis also shows that SVM and IBL models produce significantly higher sensitivity scores than SMOTE - KNN, with an average sensitivity score of 0.664 and 0.734, respectively, while SMOTE - KNN averages 0.640 across all 12 datasets. However, there are no other significant differences in the specificity performances of the machine learning methods. The only significant difference in both sensitivity and specificity detected by the Nemenyi tests is due to the stronger performance of the FLR implementations with and without SMOTE.

Table 8 presents the results for F1 performance across the FLR and machine learning models. We see a similar trend with sensitivity and specificity scores. FLR averages at 0.860 for F1 across the 12 datasets, while none of the machine learning models reach such a high F1 score. We observe that SVM, ANN, KNN, XGM, CLR, and RF reach an F1 score greater than 0.8 for breast cancer, hepatitis and heart failure datasets, but for the other six datasets, the scores are very low ($<0.66$). This lack of consistency in these models is also seen when we apply SMOTE. However, FLR achieves an even higher average F1 score of 0.908 when combined with SMOTE. There is a significant difference between the F1 scores of the compared models according to the Friedman test at 5% level of significance, with $Q=66.964, F_f=10.568, P<0.001$, while Nemenyi post hoc tests, in Table S3 of SM, indicate that both FLR implementations with and without SMOTE significantly differ from all machine learning models with and without SMOTE application. Moreover, looking into the machine learning models specifically, SMOTE - KNN shows significant variation in F1 scores against all machine learning models except ANN and SVM. KNN implementations show much lower F1 scores than not only FLR but all other machine learning models as well. XGB produces the highest F1 scores among the machine learning models, averaging 0.678. However, it is still not close to the much higher range of the FLR implementations’ F1 scores.

**Table 8:** F1 results of fuzzy logistic regression against machine learning models

	Without SMOTE							With SMOTE							
Dataset	FLR	SVM	ANN	KNN	RF	XGB	CLR	FLR	SVM	ANN	KNN	RF	XGB	CLR	IBL
Breast Cancer	0.818	0.844	0.832	0.822	0.841	0.818	0.826	0.938	0.718	0.672	0.706	0.766	0.735	0.716	0.766
Breast Cancer Wisconsin (Prognostic)	0.830	0.863	0.849	0.845	0.871	0.876	0.839	0.976	0.782	0.770	0.660	0.782	0.761	0.764	0.782
Breast Cancer Wisconsin (Diagnostic)	0.813	0.981	0.958	0.946	0.970	0.977	0.946	0.973	0.973	0.965	0.937	0.969	0.969	0.938	0.969
Hepatitis	0.801	0.889	0.877	0.866	0.902	0.885	0.886	0.942	0.882	0.779	0.676	0.880	0.834	0.783	0.880
Pima Indians Diabetes Database	0.825	0.830	0.830	0.784	0.807	0.801	0.818	0.866	0.660	0.670	0.667	0.730	0.719	0.675	0.730
Liver Disorders	0.839	0.580	0.575	0.586	0.641	0.644	0.610	0.968	0.606	0.612	0.602	0.661	0.669	0.633	0.662
SPECTF Heart	0.898	0.125	0.000	0.368	0.252	0.378	0.433	0.934	0.531	0.397	0.476	0.531	0.509	0.408	0.533
Fertility	0.927	0.000	0.000	0.000	0.000	0.090	0.022	0.883	0.142	0.114	0.208	0.200	0.191	0.242	0.200
Diabetic Retinopathy Debrecen	0.894	0.691	0.755	0.647	0.670	0.677	0.747	0.866	0.701	0.740	0.621	0.685	0.697	0.748	0.686
Breast Cancer Coimbra	0.926	0.682	0.510	0.397	0.655	0.660	0.665	0.855	0.700	0.614	0.514	0.655	0.672	0.657	0.655
Parkinson’s Disease	0.898	0.024	0.304	0.348	0.449	0.472	0.305	0.843	0.417	0.469	0.464	0.506	0.498	0.508	0.507
Heart failure	0.846	0.858	0.837	0.770	0.883	0.875	0.870	0.850	0.815	0.499	0.545	0.868	0.865	0.818	0.868

**Table 9:** MCC results of fuzzy logistic regression against machine learning models

	Without SMOTE							With SMOTE							
Dataset	FLR	SVM	ANN	KNN	RF	XGB	CLR	FLR	SVM	ANN	KNN	RF	XGB	CLR	IBL
Breast Cancer	0.725	0.269	0.304	0.202	0.326	0.289	0.305	0.845	0.210	0.148	0.192	0.270	0.220	0.273	0.270
Breast Cancer Wisconsin (Prognostic)	0.795	0.115	0.037	-0.024	0.260	0.417	0.412	0.829	0.277	0.257	0.125	0.262	0.233	0.248	0.262
Breast Cancer Wisconsin (Diagnostic)	0.788	0.948	0.885	0.851	0.917	0.937	0.856	0.852	0.927	0.905	0.828	0.918	0.917	0.836	0.918
Hepatitis	0.798	0.307	0.096	-0.036	0.423	0.388	0.417	0.852	0.471	0.236	0.161	0.449	0.369	0.220	0.449
Pima Indians Diabetes Database	0.756	0.000	0.000	0.112	0.203	0.166	0.168	0.794	0.293	0.305	0.221	0.324	0.234	0.328	0.322
Liver Disorders	0.756	0.367	0.338	0.353	0.424	0.401	0.374	0.827	0.298	0.307	0.304	0.374	0.395	0.326	0.376
SPECTF Heart	0.842	0.078	0.000	0.240	0.220	0.260	0.289	0.847	0.412	0.229	0.353	0.412	0.383	0.277	0.414
Fertility	0.756	0.000	-0.023	0.000	-0.023	0.064	-0.036	0.830	0.016	-0.066	0.045	0.069	0.037	0.123	0.069
Diabetic Retinopathy Debrecen	0.816	0.385	0.520	0.303	0.356	0.379	0.498	0.860	0.396	0.490	0.258	0.349	0.396	0.488	0.351
Breast Cancer Coimbra	0.841	0.451	0.201	-0.012	0.428	0.437	0.428	0.854	0.482	0.342	0.115	0.392	0.459	0.411	0.392
Parkinson’s Disease	0.786	0.011	0.182	0.270	0.363	0.335	0.320	0.812	0.168	0.266	0.250	0.319	0.304	0.306	0.321
Heart failure	0.824	0.534	0.331	0.016	0.626	0.599	0.589	0.856	0.488	0.279	-0.066	0.641	0.615	0.516	0.641

Table 9 presents the results for MCC. While FLR and SMOTE - FLR average 0.790 and 0.838, respectively, we see all machine learning models produce much lower MCC scores for most datasets. The average MCC score for machine learning models is 0.297 without SMOTE and improves to 0.348 with SMOTE across all datasets. Friedman test for MCC scores shows a significant difference between the compared models at a 5% level of significance with $Q=109.101, F_f=38.620, P<0.001$. Nemenyi tests, in Table S4 of SM, indicate that the significant difference between the MCCs is due to FLR being significantly different from the other models.

When the performance results in Tables 6 to 9 are considered, FLR has the minimum impact from the imbalance and separation. Across the cross-validation runs, for both FLR and machine learning models, we see an improvement when SMOTE is applied to the data. However, even with SMOTE, all machine learning models show inconsistent results between the different datasets, with performance varying based on the level of imbalance and separation present. On the other hand, FLR performs consistently well across all datasets and provides a statistically significant improvement in classification performance against machine learning models.

### Interpretation of coefficient estimates

Classifying subjects into binary categories is a pivotal task in classification problems. However, we can gain additional insights into the relative impact of each predictor in the model by using odds ratios. The fuzzy logistic regression framework applied in this study produces TFN coefficients. To interpret these, we defuzzify them using the center of gravity approach, which applies equal weighting of each of the three elements in a TFN, taking the average of these elements as the crisp representation of the TFN value. Hence, these crisp coefficients are interpreted straightforwardly in a similar manner as in classical logistic regression.

Table 10 shows the largest crisp coefficient estimate for each dataset. These are extracted by investigating all defuzzified TFN coefficients produced from the fuzzy logistic regression model. The odds ratio, $\theta$, is calculated for each crisp estimate, $\beta$, by $\theta =\exp (\beta )$.

**Table 10:** Largest coefficient estimates and their respective odds ratio, by dataset

Dataset	Predictor	TFN Value	Crisp estimate	Odds ratio
Breast Cancer	X4	(-1.796, -1.308, -0.593)	-1.232	0.292
Breast Cancer Wisconsin (Prognostic)	X12	(1.507, 1.642, 1.779)	1.643	5.171
Breast Cancer Wisconsin (Diagnostic)	X28	(1.022, 1.492, 1.645)	1.386	3.999
Hepatitis	Varices	(-1.935, -1.595, -0.582)	-1.371	0.254
Pima Indians Diabetes Database	X1	(-1.772, -0.803, 0.131)	-0.815	0.443
Liver Disorders	alkphos	(-1.087, -1.072, -0.919)	-1.026	0.358
SPECTF Heart	F9S	(-1.973, -1.403, -1.384)	-1.587	0.205
Fertility	X3	(-1.939, -1.656, 0.244)	-1.117	0.327
Diabetic Retinopathy Debrecen Dataset	X15	(1.068, 1.885, 1.995)	1.649	5.202
Breast Cancer Coimbra	HOMA	(-1.567, -1.400, -0.780)	-1.249	0.287
Parkinson’s Disease Classification	tqwt mean dec-30	(1.715, 1.733, 1.974)	1.807	6.092
Heart failure clinical records	diabetes	(-1.808, -0.831, -0.530)	-1.056	0.348

The first insight we derive from these coefficient estimates is that they all cluster to relatively small values of between $[-1.6,1.9]$. A model impacted by separation or imbalance issues often results in large coefficient estimates in absolute value, leading to odds ratios approaching infinity or a close proximity of zero [9]. However, none of the odds ratios produce values that deviate towards infinity.

Table 10 also depicts the most influential predictor in each dataset. The smallest coefficient estimate is produced for the SPECTF heart dataset for the F9S predictor, equal to $-1.587$. The X4 predictor in the breast cancer dataset, the varices predictors in the hepatitis dataset, X1 in Pima Indians diabetes data, alkphos in the liver disorders dataset, X3 in the fertility data, the HOMA predictor in the breast cancer coimbra data, and finally the diabetes predictor in the heart failure clinical records dataset are the most impactful predictors on the classification towards $Y=0$ class.

On the other hand, the largest positive coefficient is produced by the tqwt mean dec-30 predictor in the Parkinson’s disease dataset with 1.807. The X28 predictor in the breast cancer Wisconsin (prognostic), X28 in the breast cancer Wisconsin (diagnostic) dataset, and X15 in the diabetic retinopathy dataset are the most influential predictors of the classification towards the $Y=1$ class.

Given that the aim of our study is to demonstrate the performance of the fuzzy logistic regression against imbalance and separation problems in clinical studies, we are not delving into the importance and impact of each predictor in each dataset. However, it is important to note that these TFN coefficients can be defuzzified and interpreted as crisp coefficients, hence easily deriving insights on which predictor produces the most significant impact in the model of predicting a medical condition.

## Discussion and conclusion

In this study, we propose using fuzzy logistic regression to handle class imbalance and complete separation in clinical studies. The results indicate consistently high performance across all of the considered twelve datasets for the performance measures of confusion matrices, sensitivity, specificity, F1 score, MCC, and precision. The lack of perfect scores across multiple instances for all datasets indicates no influence from complete separation. Small variation between specificity and sensitivity scores indicates no impact from class imbalance on classification performance.

On the other hand, the studies in the literature show the presence of perfect scores, with several measures resulting in scores of 1.000. For some, there is a significant variation between sensitivity and specificity scores, which indicates the impact of imbalance. While there are some promising results in the reviewed literature, we have identified a significant likelihood of these results being affected by separation, imbalance, or both. However, the fuzzy logistic regression shows consistently high-performance results across all datasets with no issues due to imbalance or separation.

In a broader sense, computational efficiency with full datasets can become a challenge for the fuzzy logistic regression framework for extremely large datasets, such as Parkinson’s disease with 754 predictors, which took 61.7 hours to compile. In comparison, for the eleven datasets, which range from just seven predictors to 34, the fuzzy logistic regression framework takes an average of 59 minutes to compile, irrespective of sample size, which ranges from just $N=100$ to $N=1151$. Nevertheless, the likelihood of working with datasets of as many as 750 predictors is small compared to the potentially significant need for larger sample sizes.

Another limitation of this work is the consideration of feature selection methods along with the fuzzy logistic regression framework. In the literature, the benefits of feature selection on the performance of logistic regression are shown for classification with large datasets such as gene expression data [42–44]. A feature selection approach can be considered within the fuzzy logistic regression framework to improve the classification performance and remediate the impact of separation and the limitation on computational efficiency.

Since the codes to reproduce the results are not generally provided, it is not possible to compare the computational efficiency of the methods in the literature and the fuzzy logistic regression. It is difficult to assess how complex or efficient such methodologies truly are. On the other hand, the availability of the fuzzy logistic regression code and the ease of implementation in any given clinical study ensure strong generalizability for our results.

The fuzzy logistic regression framework can also benefit from investigating how it could be adjusted to different types of fuzzy numbers instead of just TFN. Experimenting with different types and shapes of fuzzy numbers could also result in even greater classification performance outcomes. For example, trapezoidal or Gaussian fuzzy numbers could provide a better fit in certain clinical studies, or Bayesian optimization methods can be utilized. Another area of further research is the defuzzification of coefficient estimates for crisp interpretation. While several methods can be used to defuzzify TFN coefficients, the process results in some loss of information. Further research in this field could focus on how different approaches to defuzzifying TFN coefficient estimates affect the loss of information.

In conclusion, fuzzy logistic regression offers key benefits on classification performance against class imbalance and complete separation, with strong, consistent performance across various clinical studies. There is minimal computational complexity, which ensures the generalizability of this method in different clinical studies, while the implementations on the considered datasets also show strong computational efficiency.

### Supplementary Information

Supplementary Material 1: Caption in Brief: Nemenyi post hoc test results.

## Data Availability

The datasets generated and/or analyzed during the current study are available in the UCI Machine Learning Repository repository, [https://archive.ics.uci.edu/datasets].
