# Supplementary material for: Binary classification with fuzzy logistic regression under class imbalance and complete separation in clinical studies
Source: BMC Med Res Methodol. 2024 Jul 5;24:145. doi: 10.1186/s12874-024-02270-x (PMC11225249; doi:10.1186/s12874-024-02270-x)
Supplement: Supplementary file 1 — Supplementary Material 1: Caption in Brief: Nemenyi post hoc test results. [file 12874_2024_2270_MOESM1_ESM.pdf]

Supplementary Material for Binary classification with fuzzy logistic regression under class imbalance and complete separation in clinical studies

Georgios Charizanos[1], Haydar Demirhan[1*], Duygu İçen[2]

[1*]Mathematical Sciences, School of Science, RMIT University, La Trobe St, Melbourne, 3000, Victoria, Australia.
[2]Department of Statistics, Hacettepe University, Çankaya, Ankara, 06800, Ankara, Turkiye.

*Corresponding author(s). E-mail(s): haydar.demirhan@rmit.edu.au;
Contributing authors: g.charizanos@gmail.com;
duyguicn@hacettepe.edu.tr;

Nemenyi post hoc test results for the comparison of FLR model against machine learning models

Tables S1 to S4 show the results of the Nemenyi post hoc test for sensitivity, specificity, F1 and MCC performance scores of FLR model against machine learning models.

Table S1: Nemenyi post hoc test for Sensitivity performance scores of FLR models against machine learning models (CD=4.166).

	**Without SMOTE**							**With SMOTE**							
	FLR	SVM	ANN	KNN	RF	XGB	CLR	FLR	SVM	ANN	KNN	RF	XGB	CLR	IBL
FLR	-	1.417	2.250	3.250	1.833	2.250	2.667	3.833	2.583	4.750*	5.583*	1.583	3.250	3.417	1.333
SVM	1.417	-	0.833	1.833	0.417	0.833	1.250	6.417*	1.167	3.333	4.167*	0.167	1.833	2.000	0.083
ANN	2.250	0.833	-	1.000	0.417	0.000	0.417	6.083*	0.333	2.500	3.333	0.667	1.000	1.167	0.917
KNN	3.250	1.833	1.000	-	1.417	1.000	0.583	7.083*	0.667	1.500	2.333	1.667	0.000	0.167	1.917
RF	1.833	0.417	0.417	1.417	-	0.417	0.833	5.667*	0.750	2.917	3.750	0.250	1.417	1.583	0.500
XGB	2.250	0.833	0.000	1.000	0.417	-	0.417	6.083*	0.333	2.500	3.333	0.667	1.000	1.167	0.917
CLR	2.667	1.250	0.417	0.583	0.833	0.417	-	6.500*	0.083	2.083	2.917	1.083	0.583	0.750	1.333
SMOTE-FLR	3.833	5.250*	6.083*	7.083*	5.667*	6.083*	6.500*	-	6.417*	8.583*	9.417*	5.417*	7.083*	7.250*	5.167*
SMOTE-SVM	2.583	1.167	0.333	0.667	0.750	0.333	0.083	6.417*	-	2.167	3.000	1.000	0.667	0.833	1.250
SMOTE-ANN	4.750*	3.333	2.500	1.500	2.917	2.500	2.083	8.583*	2.167	-	0.833	3.167	1.500	1.333	3.417
SMOTE-KNN	5.583*	4.167*	3.333	2.333	3.750	3.333	2.917	9.417*	3.000	0.833	-	4.000	2.333	2.167	4.250*
SMOTE-RF	1.583	0.167	0.667	1.667	0.250	0.667	1.083	5.417*	1.000	3.167	4.000	-	1.667	1.833	0.250
SMOTE-XGB	3.250	1.833	1.000	0.000	1.417	1.000	0.583	7.083*	0.667	1.500	2.333	1.667	-	0.167	1.917
SMOTE-CLR	3.417	2.000	1.167	0.167	1.583	1.167	0.750	7.250*	0.833	1.333	2.167	1.833	0.167	-	2.083
IBL	1.333	0.083	0.917	1.917	0.500	0.917	1.333	5.167*	1.250	3.417	4.250*	0.250	1.917	2.083	-

Table S2: Nemenyi post hoc test for Specificity performance scores of FLR models against machine learning models (CD=4.166).

	**Without SMOTE**							**With SMOTE**							
	FLR	SVM	ANN	KNN	RF	XGB	CLR	FLR	SVM	ANN	KNN	RF	XGB	CLR	IBL
FLR	-	3.500	3.833	5.750*	2.917	3.167	3.250	2.083	3.667	4.167*	5.667*	3.833	3.083	4.750*	3.750
SVM	3.500	-	0.333	2.250	0.583	0.333	0.250	5.750*	0.167	0.667	2.167	0.333	0.417	1.250	0.250
ANN	3.833	0.333	-	1.917	0.917	0.667	0.583	5.917*	0.167	0.333	1.833	0.000	0.750	0.917	0.083
KNN	5.750*	2.250	1.917	-	2.833	2.583	2.500	7.833*	2.083	1.583	0.083	1.917	2.667	1.000	2.000
RF	2.917	0.583	0.917	2.833	-	0.250	0.333	5.000*	0.750	1.250	2.750	0.917	0.167	1.833	0.833
XGB	3.167	0.333	0.667	2.583	0.250	-	0.083	5.250*	0.500	1.000	2.500	0.667	0.083	1.583	0.583
CLR	3.250	0.250	0.583	2.500	0.333	0.083	-	5.333*	0.417	0.917	2.417	0.583	0.167	1.500	0.500
SMOTE-FLR	2.083	5.583*	5.917*	7.833*	5.000*	5.250*	5.333*	-	5.750*	6.250*	7.750*	5.917*	5.167*	6.833*	5.833*
SMOTE-SVM	3.667	0.167	0.167	2.083	0.750	0.500	0.417	5.750*	-	0.500	2.000	0.167	0.583	1.083	0.083
SMOTE-ANN	4.167*	0.667	0.333	1.583	1.250	1.000	0.917	6.250*	0.500	-	1.500	0.333	1.083	0.583	0.417
SMOTE-KNN	5.667*	2.167	1.833	0.083	2.750	2.500	2.417	7.750*	2.000	1.500	-	1.833	2.583	0.917	1.917
SMOTE-RF	3.833	0.333	0.000	1.917	0.917	0.667	0.583	5.917*	0.167	0.333	1.833	-	0.750	0.917	0.083
SMOTE-XGB	3.083	0.417	0.750	2.667	0.167	0.083	0.167	5.167*	0.583	1.083	2.583	0.750	-	1.667	0.667
SMOTE-CLR	4.750*	1.250	0.917	1.000	1.833	1.583	1.500	6.833*	1.083	0.583	0.917	0.917	1.667	-	1.000
IBL	3.750	0.250	0.083	2.000	0.833	0.583	0.583	5.833*	0.083	0.417	1.917	0.083	0.083	0.667	-

Table S3: Nemenyi post hoc test for F1 performance scores of FLR models against machine learning models (CD=4.166).

	Without SMOTE							With SMOTE							
	FLR	SVM	ANN	KNN	RF	XGB	CLR	FLR	SVM	ANN	KNN	RF	XGB	CLR	IBL
FLR	-	1.667	3.833	5.417*	1.583	1.000	2.083	3.250	3.250	5.500*	6.750*	1.500	2.500	3.833	1.250
SVM	1.667	-	2.167	3.750	0.083	0.667	0.417	6.500*	1.583	3.833	5.083*	0.167	0.833	2.167	0.417
ANN	3.833	2.167	-	1.583	2.250	2.833	1.750	7.083*	0.583	1.667	2.917	2.333	1.333	0.000	2.583
KNN	5.417*	3.750	1.583	-	3.833	4.417*	3.333	8.667*	2.167	0.083	1.333	3.917	2.917	1.583	4.167*
RF	1.583	0.083	2.250	3.833	-	0.583	0.500	4.833*	1.667	3.917	5.167*	0.083	0.917	2.250	0.333
XGB	1.000	0.667	2.833	4.417*	0.583	-	1.083	4.250*	2.250	4.500*	5.750*	0.500	1.500	2.833	0.250
CLR	2.083	0.417	1.750	3.333	0.500	1.083	-	5.333*	1.167	3.417	4.667*	0.583	0.417	1.750	0.833
SMOTE-FLR	3.250	4.917*	7.083*	8.667*	4.833*	4.250*	5.333*	-	6.500*	8.750*	10.000*	4.750*	5.750*	7.083*	4.500*
SMOTE-SVM	3.250	1.583	0.583	2.167	1.667	2.250	1.167	6.500*	-	2.250	3.500	1.750	0.750	0.583	2.000
SMOTE-ANN	5.500*	3.833	1.667	0.083	3.917	4.500*	3.417	8.750*	2.250	-	1.250	4.000	3.000	1.667	4.250*
SMOTE-KNN	6.750*	5.083*	2.917	1.333	5.167*	5.750*	4.667*	10.000*	3.500	1.250	-	5.250*	4.250*	2.917	5.500*
SMOTE-RF	1.500	0.167	2.333	3.917	0.083	0.500	0.583	4.750*	1.750	4.000	5.250*	-	1.000	2.333	0.250
SMOTE-XGB	2.500	0.833	1.333	2.917	0.917	1.500	0.417	5.750*	0.750	3.000	4.250*	1.000	-	1.333	1.250
SMOTE-CLR	3.833	2.167	0.000	1.583	2.250	2.833	1.750	7.083*	0.583	1.667	2.917	2.333	1.333	-	2.583
IBL	1.250	0.417	2.583	4.167*	0.333	0.250	0.833	4.500*	2.000	4.250*	5.500*	0.250	1.250	2.583	-

Table S4: Nemenyi post hocc test for MCC performance scores of FLR models against machine learning models (CD=4.166).

	Without SMOTE							With SMOTE							
	FLR	SVM	ANN	KNN	RF	XGB	CLR	FLR	SVM	ANN	KNN	RF	XGB	CLR	IBL
FLR	-	6.833*	8.250*	9.500*	4.333*	3.333	4.583*	1.250	4.833*	7.750*	9.083*	3.083	4.417*	5.500*	2.750
SVM	6.833*	-	1.417	2.667	2.500	3.500	2.250	6.083*	2.000	0.917	2.250	3.750	2.417	1.333	4.083
ANN	8.250*	1.417	-	1.250	3.917	4.917*	3.667	9.500*	3.417	0.500	0.833	5.167*	3.833	2.750	5.500*
KNN	9.500*	2.667	1.250	-	5.167*	6.167*	4.917*	10.750*	4.667*	1.750	0.417	6.417*	5.083*	4.000	6.750*
RF	4.333*	2.500	3.917	5.167*	-	1.000	0.250	5.583*	0.500	3.417	4.750*	1.250	0.083	1.167	1.583
XGB	3.333	3.500	4.917*	6.167*	1.000	-	1.250	4.583*	1.500	4.417*	5.750*	0.250	1.083	2.167	0.583
CLR	4.583*	2.250	3.667	4.917*	0.250	1.250	-	5.833*	0.250	3.167	4.500*	1.500	0.167	0.917	1.833
SMOTE-FLR	1.250	8.083*	9.500*	10.750*	5.583*	4.583*	5.833*	-	6.083*	9.000*	10.333*	4.333*	5.667*	6.750*	4.000
SMOTE-SVM	4.833*	2.000	3.417	4.667*	0.500	1.500	0.250	6.083*	-	2.917	4.250*	1.750	0.417	0.667	2.083
SMOTE-ANN	7.750*	0.917	0.500	1.750	3.417	4.417*	3.167	9.000*	2.917	-	1.333	4.667*	3.333	2.250	5.000*
SMOTE-KNN	9.083*	2.250	0.833	0.417	4.750*	5.750*	4.500*	10.333*	4.250*	1.333	-	6.000*	4.667*	3.583	6.333*
SMOTE-RF	3.083	3.750	5.167*	6.417*	1.250	0.250	1.500	4.333*	1.750	4.667*	6.000*	-	1.333	2.417	0.333
SMOTE-XGB	4.417*	2.417	3.833	5.083*	0.083	1.083	0.167	5.667*	0.417	3.333	4.667*	1.333	-	1.083	1.667
SMOTE-CLR	5.500*	1.333	2.750	4.000	1.167	2.167	0.917	6.750*	0.667	2.250	3.583	2.417	1.083	-	2.750
IBL	2.750	4.083	5.500*	6.750*	1.583	0.583	1.833	4.000	2.083	5.000*	6.333*	0.333	1.667	2.750	-
